# Effects of proteases on L-glutamic acid fermentation

**DOI:** 10.1080/21655979.2019.1688224

**Published:** 2019-11-13

**Authors:** Xiaocui Liu

**Affiliations:** Department of Life Science of Shanxi Datong University, Datong Shanxi, China

**Keywords:** L-glutamic acid fermentation, hetero proteins, trypsin, degree of enzymatic hydrolysis, volumetric dissolved oxygen coefficient

## Abstract

In the process of L-glutamic acid fermentation, there are proteins that cannot be decomposed and utilized by bacteria and that secreted by bacteria at the same time, which cause problems such as increased foam production in the fermentation broth that lowers the dissolved oxygen, which makes the total fermentation efficiency low. Therefore, these proteins can be decomposed by adding proteases in the fermentation broth, and it is found that the best results are obtained by adding 0.5 g/L of trypsin. Proteins can be used by bacteria after being decomposed as well. The final L-glutamic acid production in our research was 177.0 g/L, which is 14.9% more than the control fermentation (154.0 g/L). Similarly, the glucose conversion rate was 68.3%, which is an increase of 4.0% as compared to the control fermentation (65.6%).

## Graphical Abstract

**Figure:**
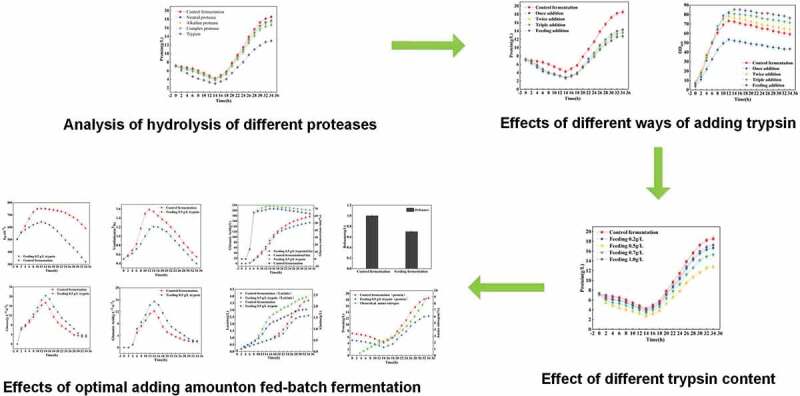

## Introduction

The production of L-glutamic acid has undergone substantial changes over time. A transformation from the hydrolysis and enzymatic hydrolysis methods to the fermentation, which has greatly improved the acid production, reduced the production cost, thus making the L-glutamic acid one of the widely used bulk amino acids [1; 2]. The main L-glutamic acid-producing bacteria are *Brevibacterium flavum, Corynebacterium glutamicum*, and *Corynebacterium sclerophylla*. Additionally, various high-efficiency industrial strains have been obtained through selective breeding and genetic engineering [3,4]. By optimizing the fermentation medium used, the control of fermentation process and product separation and extraction process, the yield of L-glutamic acid has been further increased and the production cost has been reduced [5]. For L-glutamic acid production, the use of nutrient-rich and inexpensive fermentation media is one of the main strategies for reducing production costs [6]. Therefore, the main sources of L-glutamic acid fermentation nutrients include corn syrup, soybean meal hydrolyzate. The corn syrup and the soybean meal hydrolyzate contain a variety of small molecular weight proteins, peptides, and a variety of amino acids. They also contain a wide range of vitamins and trace elements, which are indispensable for the growth of the cells [7,8]. These substances are used as nitrogen sources and cofactors for cell growth and greatly increase enzymatic activity of bacteria, which provide a stable foundation for a good fermentation [9]. However, the corn syrup and soybean meal hydrolyzate also contain a large number of macromolecular proteins that are difficult to be decomposed and utilized by the bacteria. These proteins not only produce foam during the fermentation process, thus lowering the dissolved oxygen efficiency and mass transfer, but also produce more waste and reduce the amount of medium available for bacterial growth. The utilization efficiency also increases the difficulty of separation and extraction of the fermentation product. On the other hand, most of the engineered bacteria themselves produce a large amount of extracellular proteins in the fermentation process, especially in the middle and late stages of fermentation. These proteins cause waste of raw materials and reduce the glucose conversion rate leading to the problems mentioned above.

To overcome these complications, this study adopts a fermentation process in which proteases are added [10,11]. By studying different kinds of proteases, its addition amount and method, trypsin was finally selected as the best choice [12–14]. Trypsin addition during the fermentation process can hydrolyze a variety of extracellular proteins into amino acids and small peptides, which not only solve a series of fermentation problems caused by proteins, but also increase the utilization of protein hydrolyzates by the bacteria [15–18]. An efficient use of the medium and together with optimal production of the bacterial metabolites were achieved. Although trypsin addition cannot completely decompose all the proteins in the medium, it hydrolyzes a considerable amount of them and has a positive effect on the fermentation of L-glutamic acid [19].

## Materials and methods

### Strains

*Brevibacterium flavum GDK-168* (suboptimal biotin high-yield strains) [20].

### Cultivation methods

#### Culture medium

##### Activated slant medium (g/L)

Beef extract 10.0, peptone 5.0, yeast extract powder 5.0, KH_2_PO_4_ 1.0, MgSO_4_ · 7H_2_O 0.5, corn syrup 20.0 ml/L, agar 25.0, pH7.0.

##### Strain medium (g/L)

Oral glucose 25.0, corn syrup 33.0 ml/L, soybean meal hydrolyzate 22.0 ml/L, K_2_HPO_4_ · 3H_2_O 2.2, MgSO_4_ · 7H_2_O 1.0, methionine 2.0.

##### Fermentation medium (g/L)

Oral glucose 80.0, Na_2_HPO_4_ · 12H_2_O 3.0, MgSO_4_ · 7H_2_O 1.8, KCl 1.7, methionine 2.0, MnSO_4_·H_2_O 2.5 mg/L, FeSO_4_ · 7H_2_O 2.5 mg/L, VB_1_ 0.5 mg/L, molasses 1.0, corn syrup 4.0 ml/L, soybean meal hydrolyzate 20.0 ml/L.

#### Culture conditions

##### Strain activation

The glycerin tube was stored at −80°C and placed next to the alcohol flame in the sterile room. The inoculation ring was used to dip in the suspension of 2–4 rings bacteria and inoculated in the inclined surface of the test tube. The suspension was cultured at 32°C for 12 h. The bacteria in the first generation of slant were transferred to the 4 new test tube slants and cultured at 32°C for 12 h.

##### Primary seed cultivation

Four second-generation inclined planes were inoculated into four 1000 ml round bottom triangular bottles containing 100 ml medium, and cultured at 34°C at 180 r/min in a shaking table for 10 ~ 12 h.

##### Secondary seed cultivation

The primary seeds were cultured in a 5 L fermentation reactor containing 2.4 L medium under the protection of the flame circle. Maintain pH between 7.0 and 7.2 using ammonia water, When the dissolved oxygen is maintained at about 30%, the temperature is 34°C and OD_600_1.1 × 20, the seeds can be transferred to the fermentation reactor.

##### Fermentation cultivation

With an inoculation volume of about 20%, secondary seed liquid was inserted into a 30 L sterile fermentation reactor, with an initial volume of 14 L, a rotation speed of 400 rpm, ventilation volume of 0.5 m^3^/h, and a pH of 7.2. During the fermentation process, the pH value was adjusted by adding ammonia water, and the dissolved oxygen content was maintained by adjusting the rotational speed and ventilation rate. Dissolved oxygen from 0 to 12 h should be controlled in the range of 20% to 40%, from 12 to 14 h should be controlled in the range of 15% to 20%, and from 14 h to 15%. The initial fermentation temperature was 34°C, every 4 h, increase 0.5°C until 36°C (16 h) and remain unchanged until the end of fermentation. The fermentation time was 34 ~ 36 h.

### Data analysis

All experimental data were averaged from the 3 experiments. After one-way anova, dunnet t test was performed to determine the significance of the data difference (P < 0.05).

### Experimental methods

#### Basic properties of different proteases

Referring Table 1.

**Table 1.** Basic properties of different proteases.

Species	Optimum temperature (°C)	Optimum pH	Enzyme activity (U/g)
Trypsin	37.0	8.0	250,000.0
Neutral protease	45.0 ~ 50.0	6.8 ~ 7.0	200,000.0
Alkaline protease	40.0 ~ 50.0	9.0 ~ 12.0	200,000.0
Complex flavor protease	53.0	6.0 ~ 7.0	250,000.0

#### Aseptic processing of proteases

An amount of different kinds of proteases was weighed, dissolved in 50.0 or 200.0 ml of sterile water in a sterile room, and then aspirated from the solution using a 50.0 ml sterile syringe. Then, the solution was sieved through a 0.2 μm pore size microfiltration membrane. The so obtained sterile enzyme solution was placed in a sterile flask or into a feeding bottle.

#### Feeding different proteases process

According to the amount of 0.5 g/L (preliminary experiment results), 10.0 g (total volume of fermentation volume of 20.0 L) of alkaline protease, neutral protease, complex flavor protease, and trypsin were weighed separately. After the above mentioned aseptic treatment, L-glutamic acid fermentation was carried out in 30.0 L tank. The feeding was started at the beginning of the fermentation and lasted until 4.0 h before the end of the fermentation.

#### Different ways of adding trypsin

After the optimal amounts of proteases to add were determined, different addition methods were performed. According to the amount of 0.5 g/L (total fermentation volume of 20.0 L), the fermentation was started in the 30.0 L fermenter, and the single addition (10.0 g), the double addition (adding 5.0 g at fermentation starts and adding 5.0 g at fermentation 14.0 h), triple addition (adding 3.0 g at fermentation starts, adding 3.0 g at fermentation 14.0 h and adding 4.0 g at fermentaton 24.0 h) and feeding (10.0 g) of fermentation.

#### Feeding different amounts of trypsin

The optimal addition method obtained by the above process was feeding trypsin; then the experiments of feeding different amounts of trypsin were carried out. According to the amounts of 0.3 g/L, 0.5 g/L, 0.7 g/L, and 1.0 g/L, 6.0 g, 10.0 g, 14.0 g, and 20.0 g trypsin were weighed and sterilized, and then fed in the fermentation in the 30.0 L fermenter. The trypsin feeding during fermentation was performed and all the feedings were completed at 4.0 h before the end of the fermentation.

### Detection methods

#### Determination of total protein content

The total proteins contained in the fermentation broth were quantitatively detected using a total proteins quantification test kit (BCA method) (Which was produced by Nanjing Jiancheng Institute of Bioengineering; its principle is by measuring absorption value).

#### Determination of total nitrogen content

The total nitrogen content in the fermentation broth was determined by Kjeldahl method [21].

#### Determination of volumetric dissolved oxygen coefficient k_l_a

The volumetric dissolved oxygen coefficient K_L_a can be calculated by the formula: $K_{L}a=\frac{F_{O_{2}}^{in}-F_{O_{2}}^{out}}{Vc^{*}\left(1-c/c^{*}\right)}$, where $F_{O_{2}}^{in}$ is the O_2_ in flow of the fermentation system, mol; $F_{O_{2}}^{out}$ is instead the O_2_ out flowing out of the fermentation system, mol; V is the volume of the fermentation liquid, L; $c^{*}$ is the saturation concentration of oxygen in water, generally taking $c^{*}$= 2 × 10^−4^ mol/L; and *c* is the real-time oxygen concentration of the fermentation system, mol/L. A dissolved oxygen electrode was used to detect changes in dissolved oxygen in the fermentation liquid, and a the tail gas analyzer was used to detect the oxygen concentration in and outside of the fermentation container. The value of K_L_a can be obtained by the above formula.

#### Determination of organic acids

The detection of organic acids in the fermentation broth was performed by using a high-performance liquid chromatography analyzer. One milliliter of the fermentation broth was added in a 1.5 mL centrifuge tube, centrifuged at 18,000 g for 2.0 min, the supernatant was diluted as appropriate and then filtered with a 0.22 μm microporous membrane. The filtrate was used as a sample for liquid chromatography. Conditions: The column used was Bio-Rad Aminex HPX-87H column (300.0 mm × 7.8 mm, 9.0 μm), eluted with 0.05 mol/L sulfuric acid, at a temperature of 30.0°C and with a flow rate of 0.5 mL/min, detection wavelength 210.0 nm.

#### Determination of amino acids

The amino acid content in the fermentation broth was determined by an amino acid analyzer. The fermentation broth was treated in the same manner as the organic acid, diluted as appropriate, and then filtered through a 0.22 μm microporous membrane filter. Filtrate was quantitatively detected using an amino acid analyzer. The chromatographic conditions used were: LCAK06/Na column (4.6 × 150.0 mm) Buffer A and Buffer B were eluted at a column temperature of 60.0°C, a flow rate of 0.45 mL/min, and detection wavelengths of 570.0 nm and 440.0 nm.

#### Determination of L-glutamic acid production and fermentation parameters

The contents of L-glutamic acid and glucose in the fermentation broth were detected by a SBA-40E biosensor analyzer; the pH and dissolved oxygen content were instead detected by pH dissolved oxygen electrodes respectively.

## Results

### Effects of different proteases on the degree of protein hydrolysis in L-glutamic acid fermentation broth

**Figure 1.**
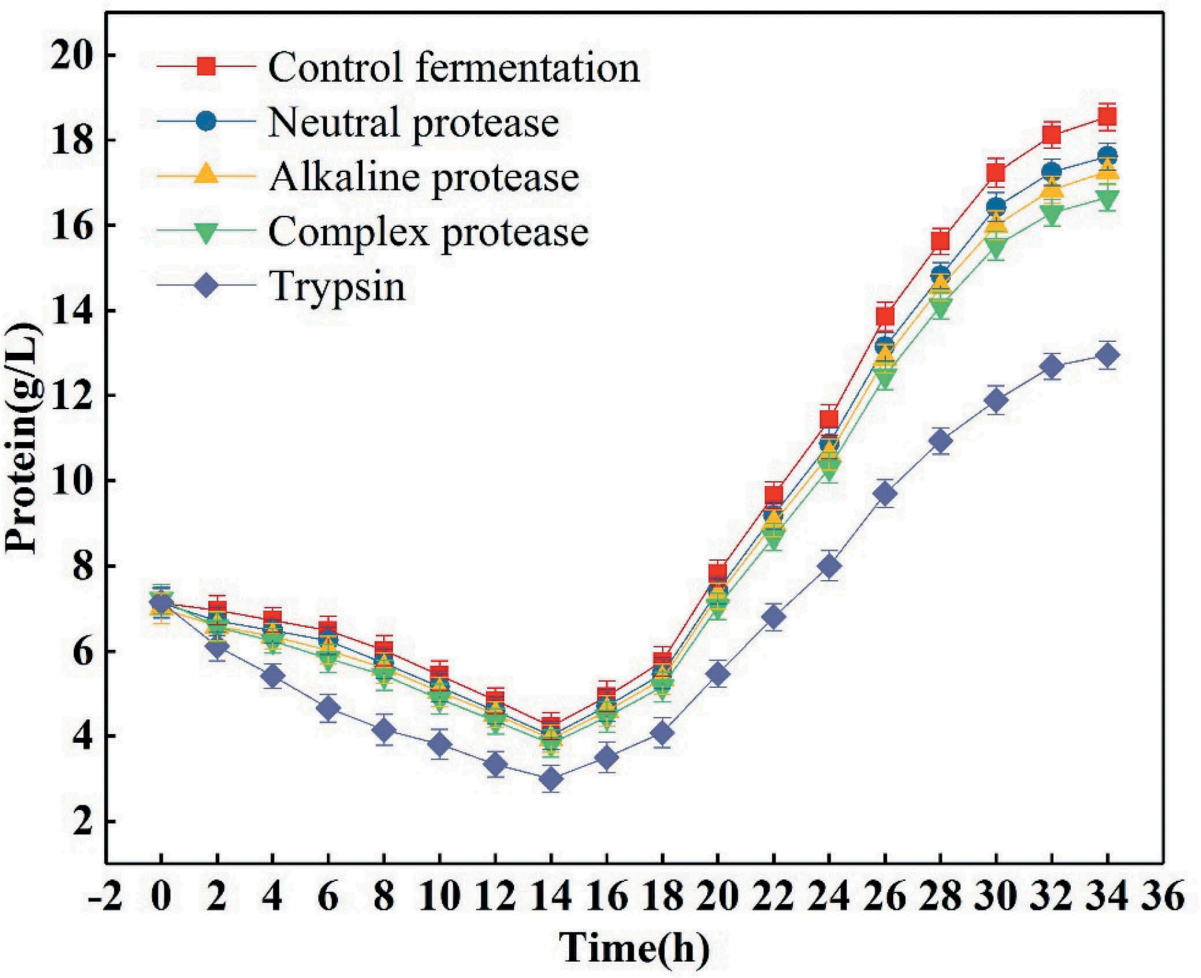
Effects of feeding 0.5 g/L different proteases on total protein content.

Owing to the enzymatic activity conditions of many proteases were far from the control conditions of the L-glutamic acid fermentation process, and were not suitable for the fermentation process [22,23]. Therefore, this study selected commonly used alkaline protease, neutral protease, complex flavor protease and trypsin as the research objects, to select the most efficient one. Firstly, the ability of different proteases to hydrolyze proteins during L-glutamic acid fermentation was studied. The initial experiment was performed by using appropriate enzyme amount (0.5 g/L) and addition mode. For control fermentation, since corn syrup, soybean meal hydrolyzate were used and seed liquid contain more proteins, the fermentation broth contains a considerable amount of proteins (about 7.12 g/L) at the time of initial fermentation. Some of the proteins in the corn syrup and soybean meal hydrolyzate can be decomposed and utilized by the bacteria, and the bacteria produce almost no heterologous proteins at early stage of fermentation, the total protein content of the fermentation broth in the early stage of fermentation shows a downward tendency [24–26]. As the fermentation enters middle and late stages, more and more heterologous proteins are formed due to the decrease of bacterial vigor and the total protein content in the fermentation broth begin to rise. The protein content in the L-glutamic acid fermentation broth are shown in Figure 1 (excluding proteins content in proteases, the below is same). It can be seen from the figure that when the proteases are added, the total protein contents rapidly decrease due to the decomposition of some proteins in the corn syrup and soybean meal hydrolyzate into various amino acids, and the total protein content are minimized after fermentation to 14 h. At the same time, as the viability of the bacteria begins to decline, the amount of production of hybrid proteins begins to increase as the L-glutamic acid fermentation conditions (pH 7.2 ~ 7.5; initial temperature of 34.0°C, increase of 0.5°C every 4 h reaching finally to 36.0°C and then maintained unchanged) are not optimal for activity of proteases. In addition, some proteins are not substrates of the proteases. Therefore, although the proteases continue to hydrolyze, the amount of proteins produced is greater than the amount of hydrolyzed, and the total protein content begins to increase. For the several proteases used in this study, the degree of hydrolysis is not the same. As compared to the control fermentation, the amount of total proteins in the fermentation broth was reduced when the protease was added, and a large portion of the proteins was hydrolyzed. It was found that trypsin was the best of the proteases used, and its degree of total protein hydrolysis was as high as 30% (compared to the total protein content of control fermentation), while that for the remaining proteases was up to 10%. Therefore, trypsin is the best choice according to this study.

### Effects of different ways of trypsin addition on the degree of protein hydrolysis in l-glutamic acid fermentation broth

**Figure 2.**
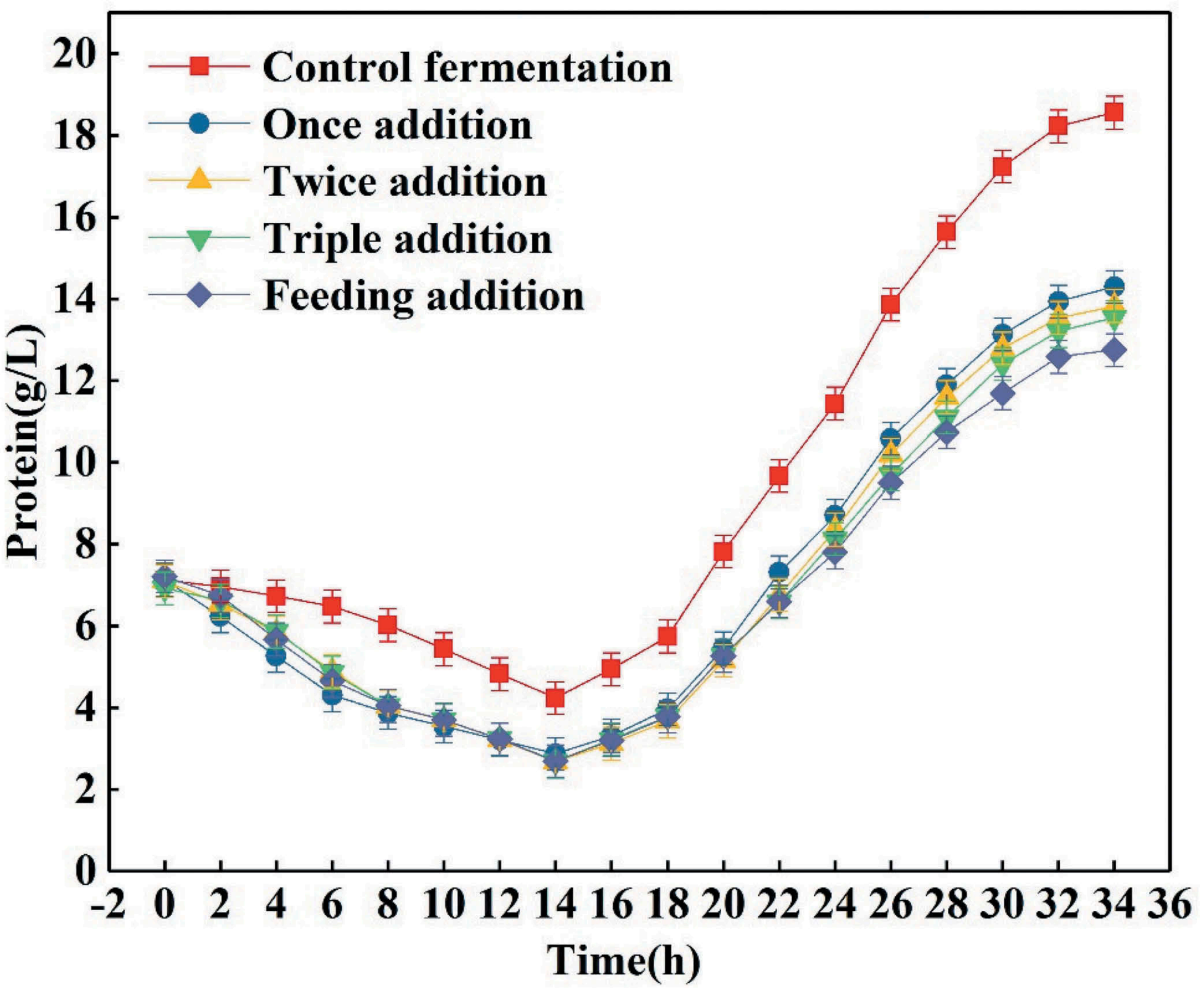
Effects of different addition methods of trypsin (0.5 g/L) on total protein content.

Different ways of adding trypsin have differences in the hydrolysis effect of proteins in the fermentation broth. In this study, according to the amount of enzyme used of 0.5 g/L (total volume of fermentation of 20.0 L), single addition (10.0 g), double addition (5.0 g for fermentation start, 5.0 g for 14.0 h), and triple addition (3.0 g for fermentation start, 4.0 g in 14.0 h, 4.0 g in 24.0 h,) and feeding (10.0 g) fermentation. Single addition means that the whole amount of trypsin is added at one time at the beginning of the fermentation. As can be seen from the result shown in Figure 2, compared with the other three methods of addition, due to the large number of enzymes, the total protein content in the early stage of fermentation decreased rapidly, and the hydrolysis effect was good. However, due to the large difference between the fermentation conditions and the optimal conditions for higher enzymatic activity, and the fact that the fermentation broth contains substances that inhibit the enzyme activity, the trypsin activity decreases rapidly and the protein hydrolysis at middle and late stages of fermentation is poor. The same problems occurred in the double triple addition groups, although the total hydrolysis effect is better than that in the single addition, mainly because the total trypsin activity can be maintained for a long time. The feeding method allows the trypsin to be continuously added to the fermentation broth. When the activity of the trypsin added in the previous period begins to decrease, a new amount of trypsin is added, so that the relative enzyme activity in the fermentation broth is always maintained high trypsin activity is capable of continuously hydrolyzing proteins for optimal hydrolysis. Therefore, it can be seen that the feeding of trypsin is the best addition mode.

### Effects of different ways of trypsin addition on the number of bacteria (OD_600_ value)

**Figure 3.**
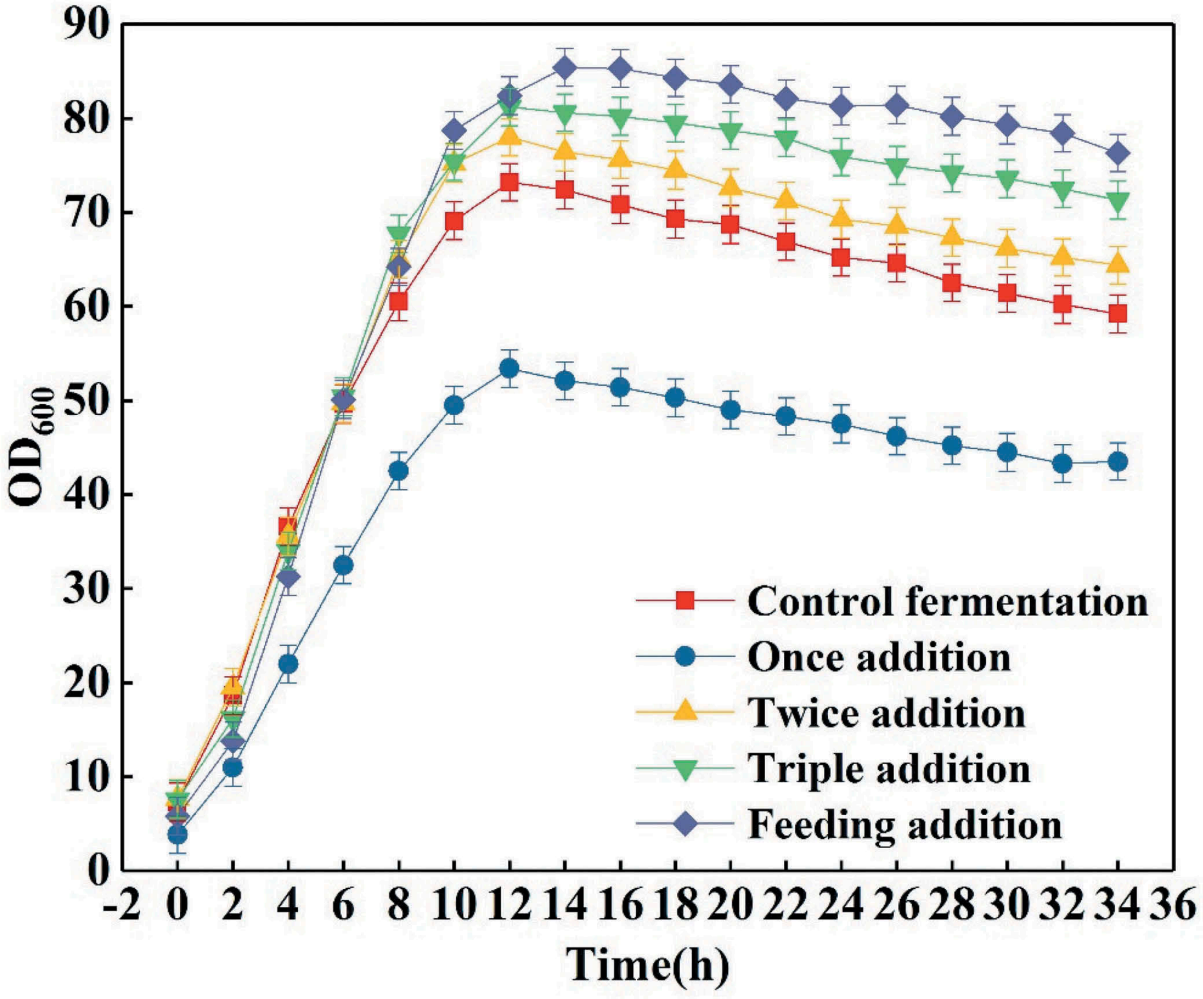
Effects of different addition methods of trypsin (0.5 g/L) on OD_600._

As can be seen from Figure 3, the effect of different trypsin addition methods on the number of bacteria (OD_600_) is also quite different. Compared with the control fermentation, double addition, triple addition, and feeding can promote the increase in the number of bacteria. This happens mainly because the proteins are hydrolyzed into various amino acids, small peptides, and small molecular proteins that will be used by the bacteria, not only for the purpose of producing the compound of interest, but also for their growth to promote cells’ enzymatic activity. In addition, for the single supplementation because of the large amount of enzyme added, the proteins in the fermentation broth will be decomposed as well as cell membrane proteins. For the same reason, the short peptides of the cell wall will be damaged to some extent, resulting in the incompleteness of the bacteria and the reduction their number. Thus, causing the activity of the cells to decrease, and finally the total biomass of the cells to diminish. By feeding trypsin, the OD_600_ of the cells was finally 85.0, which was 13.3% higher than that of the control fermentation (OD_600_ was 75.0). Therefore, the feeding of trypsin is the best addition mode.

### Effects of feeding different dosages trypsin on the degree of protein hydrolysis in l-glutamic acid fermentation broth

**Figure 4.**
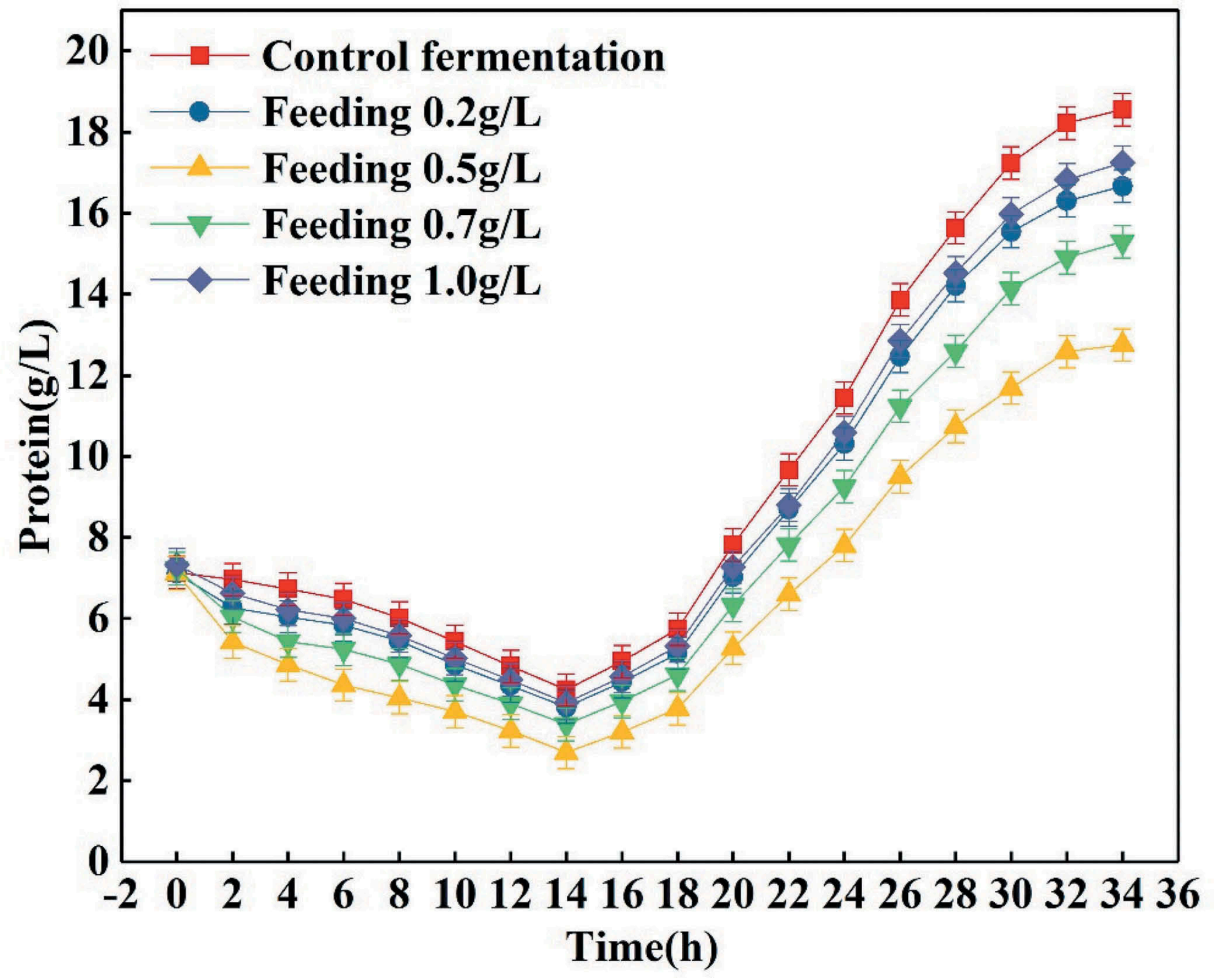
Effects of feeding different dosages trypsin on total proteins.

From the above analysis, it is known that the effect of feeding trypsin is the best, but different feeding amounts are predicted to have different effects. As can be seen in Figure 4, when the amount of feeding is 0.3 g/L, although a certain amount (about 10.0%) of the proteins can be hydrolyzed, this is not the optimal hydrolysis effect because the total amount of trypsin is small. When the amount of the feeding is 0.7 g/L, although the amount of the trypsin is sufficient to decompose heterologous proteins, the excessive content in trypsin has side effects on the cells, the enzyme activity and biomass of the cells are then lowered, and by-products which in turn inhibit the activity of trypsin are increased. Therefore, the hydrolysis effect is not good. When the amount of trypsin feeding reaches 1.0 g/L, the hydrolysis effect (about 7.0%) is greatly reduced. Therefore, excessive feeding does not achieve a good effect, but reduces the hydrolysis effect and wastes. When the amount of trypsin is 0.5 g/L, up to 30.0% of the proteins are hydrolyzed into various amino acids and small peptides. This is the best outcome for the hydrolysis of total proteins in the fermentation broth.

### Effects of feeding 0.5 g/L trypsin on the L-glutamic acid fermentation parameters

#### Effects of feeding 0.5 g/L trypsin on K_L_a and ventilation

According to the above analysis, feeding 0.5 g/L trypsin has the best hydrolysis effect on the proteins contained in the fermentation broth during L-glutamic acid fermentation. The series of changes in the fermentation process caused by the hydrolysis are mainly reflected in the fermentation process. Therefore, it is necessary to analyze this accordingly.

**Figure 5.**
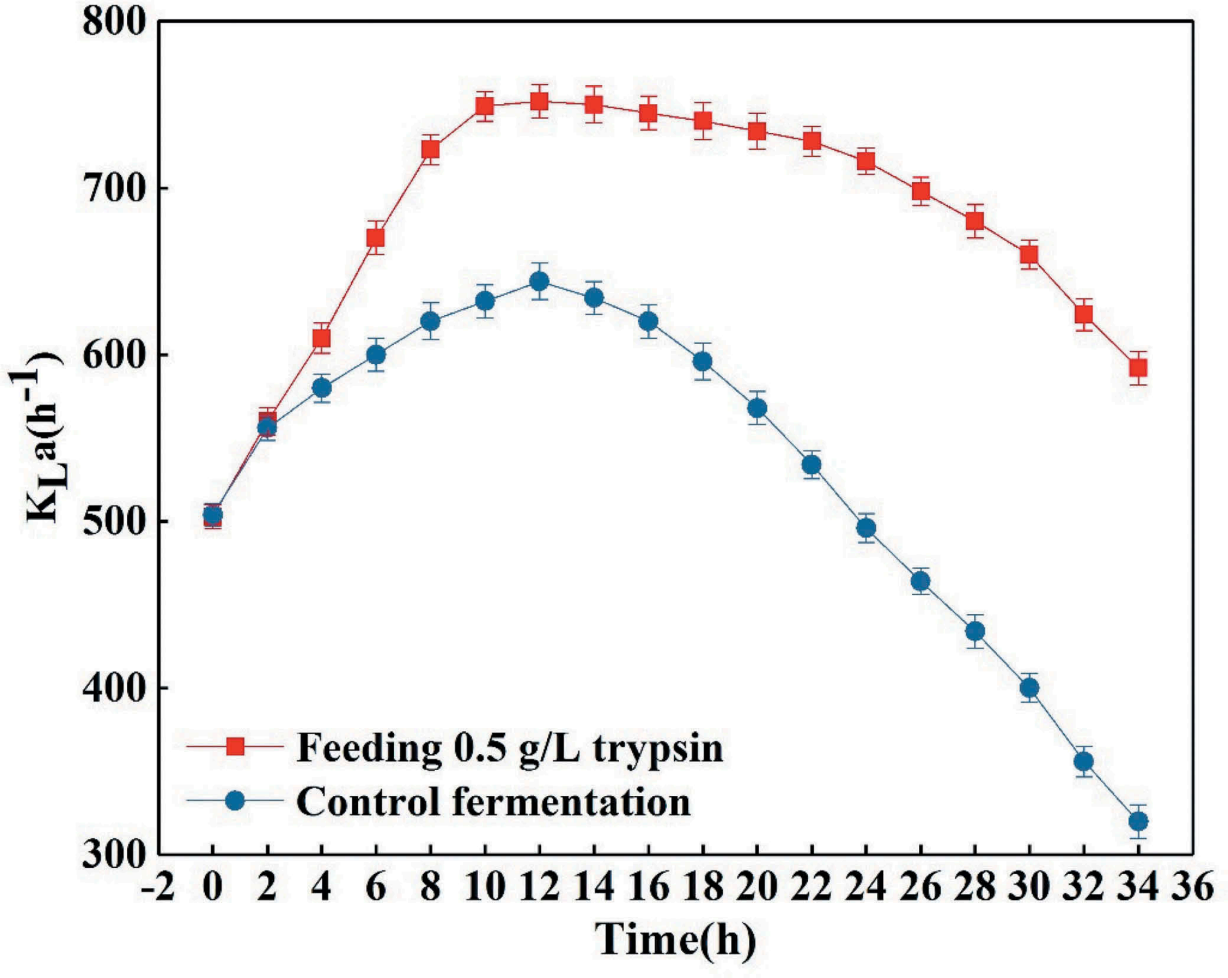
Effects of flowing 0.5 g/L trypsin on K_L_a.

**Figure 6.**
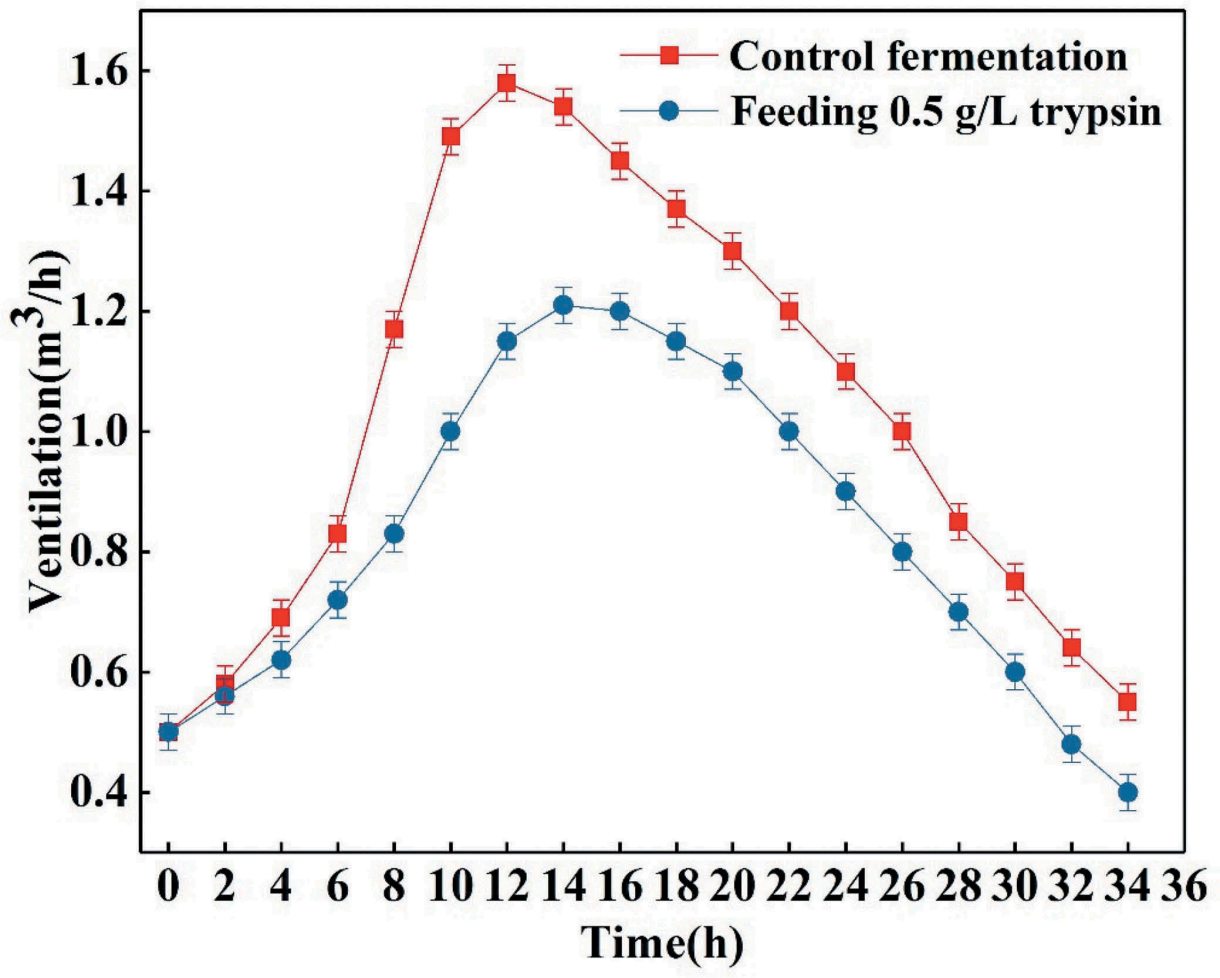
Effects of feeding 0.5 g/L trypsin on ventilation.

The heterologous proteins in the fermentation broth are hydrolyzed into various amino acids, thus reducing the viscosity of the fermentation broth. The most important effect to achieve is to greatly improve the dissolution efficiency of oxygen, which can be seen from the change of the volume dissolved oxygen coefficient. As shown in Figure 5, it can be seen that when the ventilation amount of the control fermentation and the feeding trypsin fermentation are controlled under the same conditions, the K_L_a changes. It is not difficult to find that the increase of K_L_a in the feeding trypsin fermentation is obvious. In the early stage of fermentation, due to the small number of bacteria, utilization of nutrients in the fermentation broth and the enzymatic hydrolysis, the viscosity of the fermentation broth is reduced, so the total K_L_a is on the rise. After the fermentation continued for about 12.0 h, the proteins produced by the bacteria began to increase and the total K_L_a began to slowly decrease, while the control fermentation decreased rapidly, and the total K_L_a value of the trypsin fermentation was much higher than the K_L_a value of the control one [27]. On the other hand, when the K_L_a values of the controlled feeding fermentation and the control fermentation process are similar, the amount of ventilation needed during the whole fermentation process decreases as shown in Figure 6. In Figure 6 it can be seen that the ventilation amount of the feeding fermentation is significantly lower than that of the control fermentation.

#### Effects of feeding 0.5 g/L trypsin on the rate of glucose consumption and L-glutamic acid production

**Figure 7.**
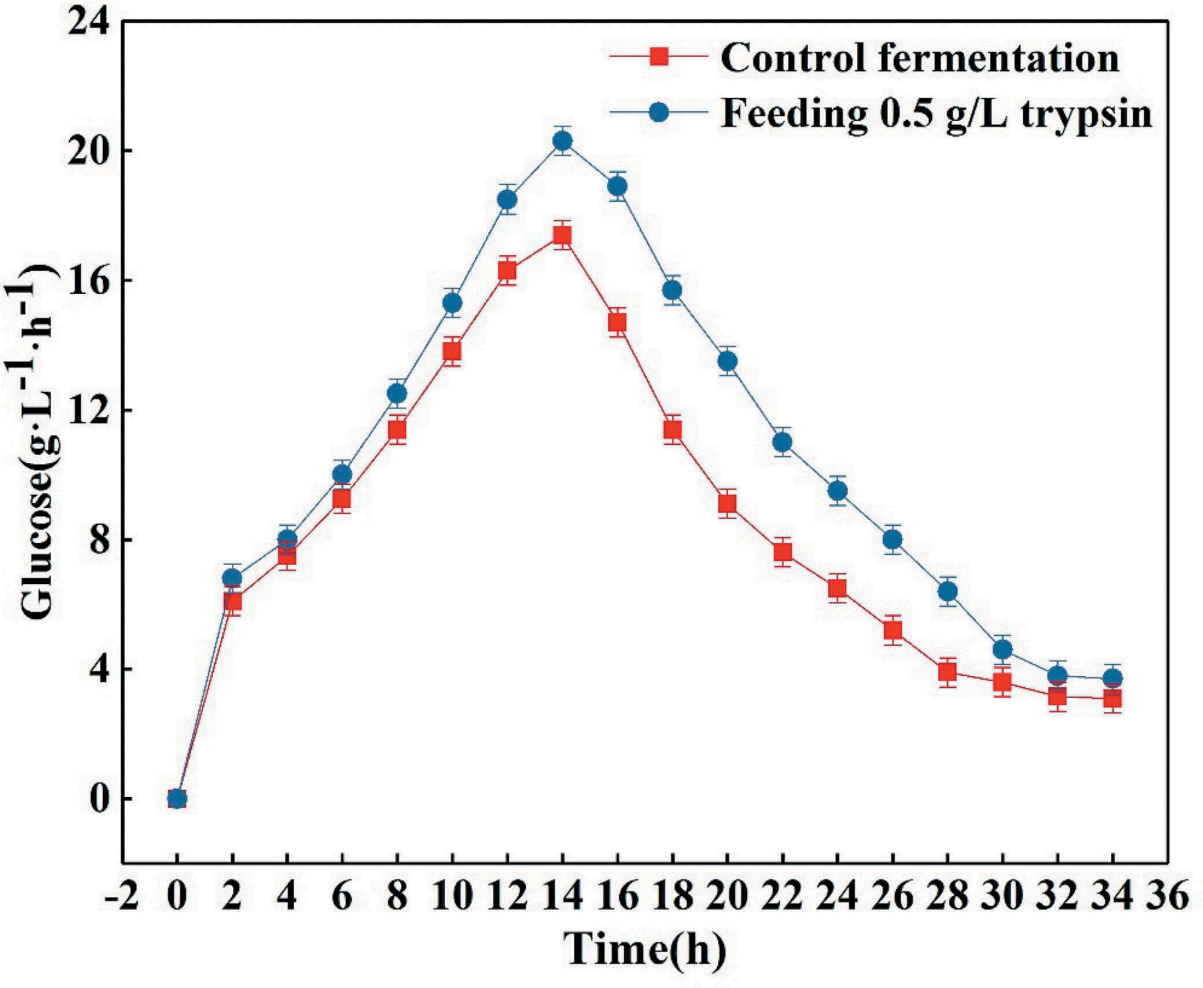
Effects of feeding 0.5 g/L trypsin on the rate of glucose consumption.

**Figure 8.**
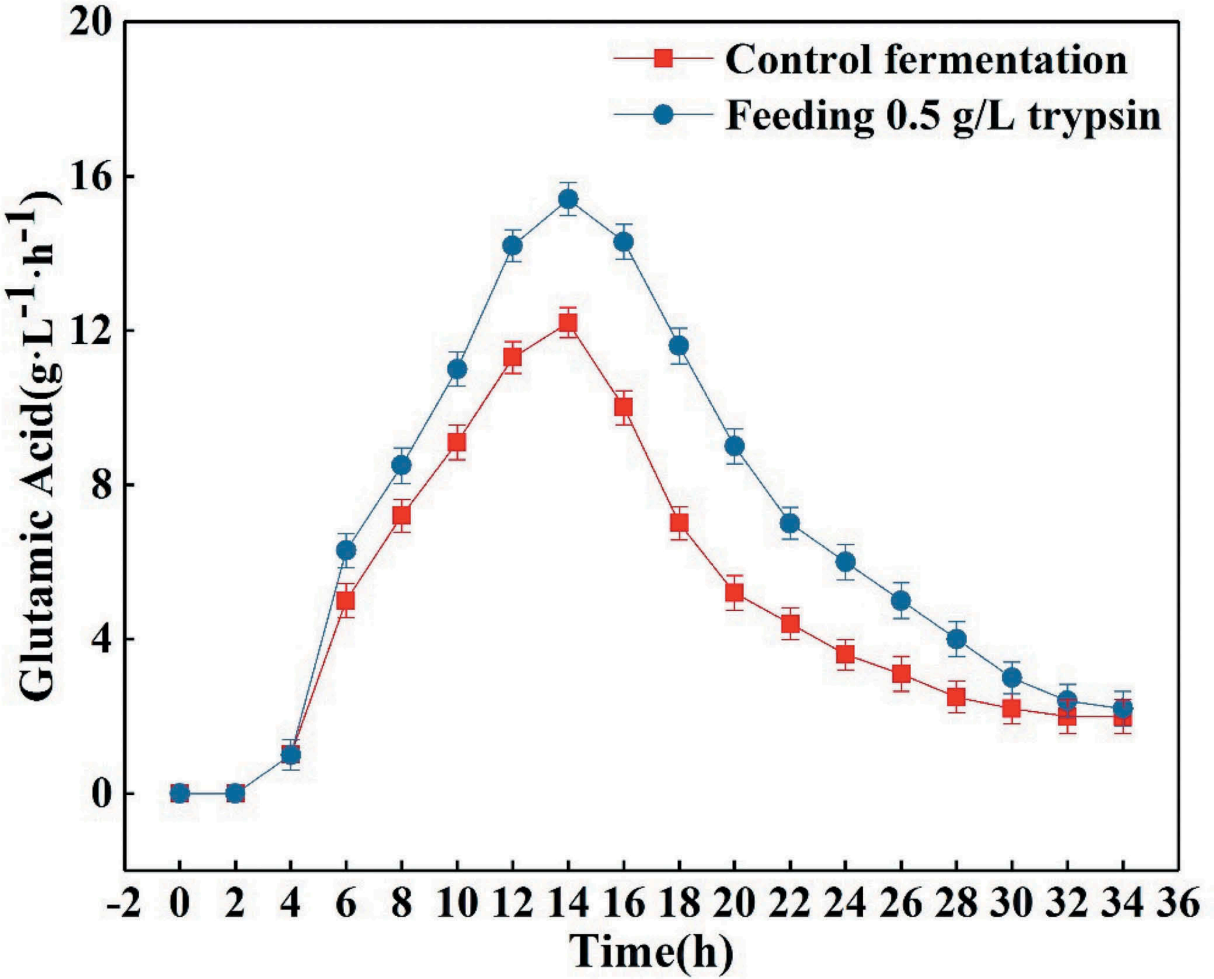
Effects of feeding 0.5 g/L trypsin on the L-glutamic acid production rate.

As shown in Figure 7, the glucose consumption rate is significantly higher when the feeding fermentation is performed as compared to the amount of glucose consumed per liter and per hour in the control fermentation. Although the glucose consumption rates of both the groups increased continuously from 2.0 to 14.0 h and the highest glucose consumption rate was reached in the trypsin group as compared to the control one, 21.5 g/(L• h) and 18.5 g/(L• h) respectively, after 14.0 h of fermentation, the decline in the rate of glucose consumption in the control fermentation was significantly higher than that the one in the feeding fermentation group, while the rate of glucose consumption in the feeding fermentation was relatively slower. The rate of glucose consumption at the same fermentation time was significantly higher in the group were trypsin was added than that in the control fermentation. However, as visible in Figure 8, the L-glutamic acid production rates were also very similar between the two analyzed groups. Before 14.0 h of fermentation, the acid production rates of both groups increased continuously, but in the feeding fermentation significantly higher values were observed as compared to the control fermentation. The L-glutamic acid production rate reached the maximum values of 16.0 g/(L• h) in the feeding fermentation and 12.5 g/(L • h) in the control one at 14.0 h. After 14.0 h, the L-glutamic acid production rate of the control fermentation decreased rapidly, and the feeding fermentation decreased relatively slow, and maintained levels that were always higher than the ones observed in the control fermentation.

#### Effects of feeding 0.5 g/L trypsin on the yield of L-glutamic acid and glucose conversion rate

**Figure 9.**
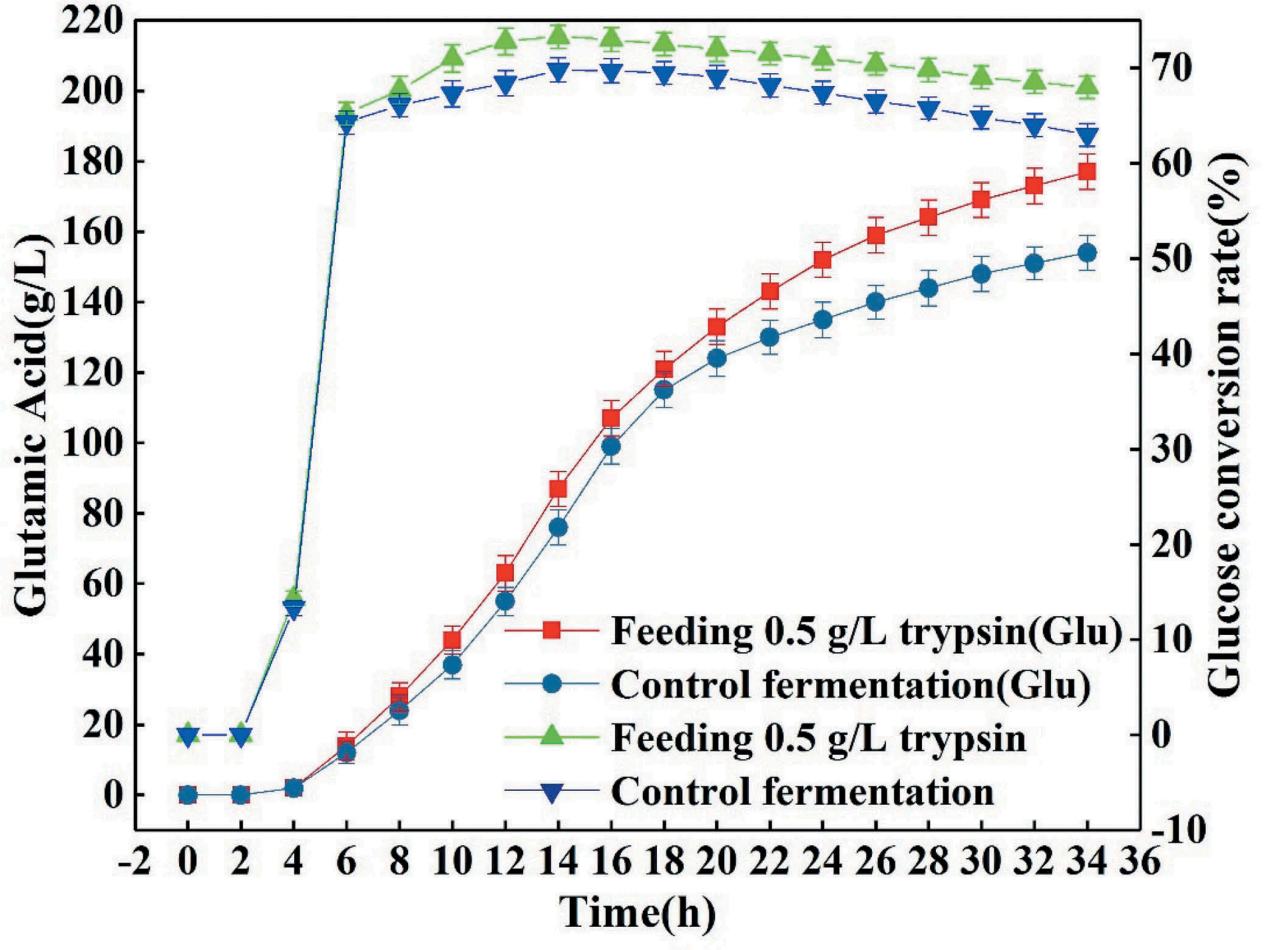
Effects of feeding 0.5 g/L trypsin on the yield of L-glutamic acid and glucose conversion rate.

It can be seen from Figure 9 that when L-glutamic acid starts to be produced after fermentation for 4 h, the total amount of L-glutamic acid in the feeding fermentation and the control fermentation increases as the fermentation progresses. However, the rate of increase of feeding fermentation was significantly higher than that of the control one, especially since the fermentation was conducted for 16 h, the rate of increase of L-glutamic acid in the control fermentation was significantly slower. In addition, the glucose conversion rate in the feeding fermentation was higher than that in the control one, and the total glucose conversion rate was also increased from 65.6% to 68.3%. Similarly, the yield of L-glutamic acid in a single tank (20.0 L of fermentation broth) was also increased from 154.0 g/L of the control fermentation to 177.0 g/L of the feeding one, an increase of 14.9%.

#### Effects of feeding 0.5 g/L trypsin on the dosage of defoamer

**Figure 10.**
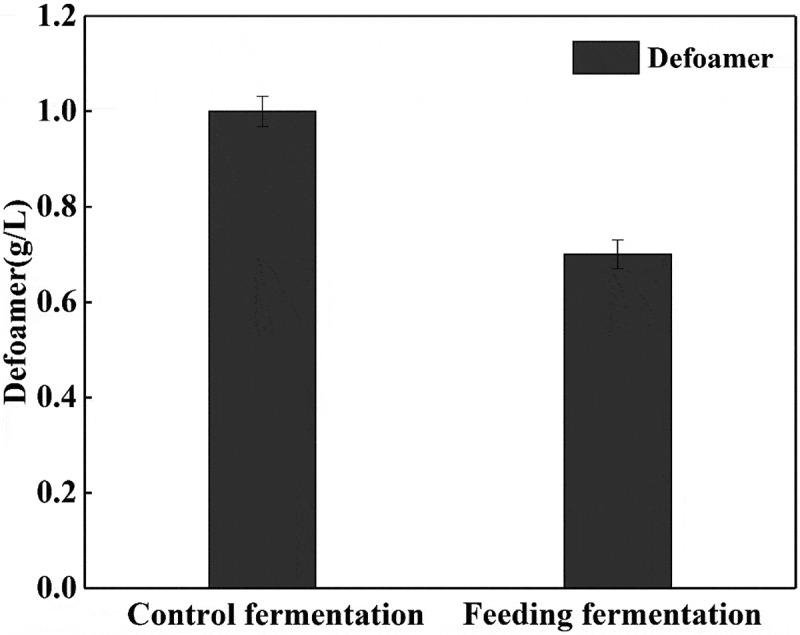
Effects of feeding 0.5 g/L trypsin on the dosage of defoamer.

Due to the large amount of foam generated during the fermentation of L-glutamic acid, excessive foam easily causes problems such as lowing dissolved oxygen efficiency and liquid medium losing [28]. Therefore, a certain amount of defoamer is used in the fermentation process, but this cannot be metabolized by the bacteria and too much addition will inhibit their growth and increase the difficulty of subsequent L-glutamic acid extraction. When feeding with trypsin, the protein content in the fermentation broth is reduced, as well as the amount of fermented foam, thus decreasing viscosity and density of the fermentation broth. As can be seen from Figure 10, the amount of defoamer was reduced from 1.0 g/L of the feeding group to 0.7 g/L of the control one.

### Effects of feeding 0.5 g/L trypsin on secondary acid production

**Figure 11.**
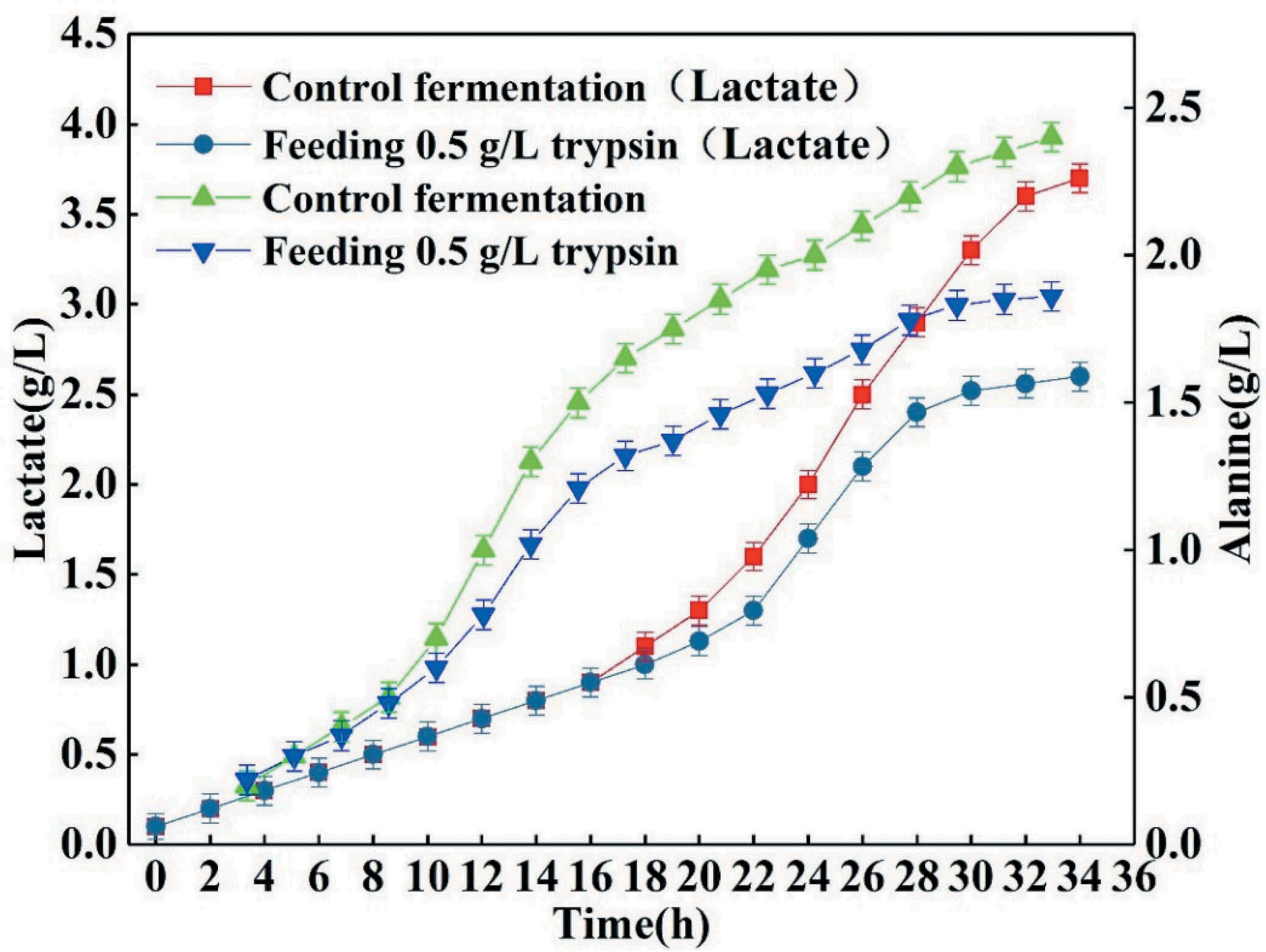
Effects of feeding 0.5 g/L trypsin on secondary acid.

For the TCA cycle, when the reducing powers NADH+H^+^ and FADH_2_ are produced in the fermentation broth during dissolved oxygen is low, the reducing power carried by the fermentation cannot use O_2_ as a hydrogen acceptor. In this case, the glycolysis pathway product pyruvic acid is used as a reduction agent, and the by-product lactic acid is produced in a large amount [29]. In addition, the amount of FAD produced is also lowered due to insufficient dissolved oxygen, which further causes a decrease in the TCA cycle and a decrease in L-glutamic acid production. At the same time, pyruvic acid also accumulates, and too much pyruvic acid will further produce the by-product alanine. When the efficiency of dissolved oxygen is improved because of the protein hydrolysis, the TCA cycle is strengthened, the amount of secondary acid is greatly reduced, and the rate of formation is also lowered. Then, the yield of L-glutamic acid is simultaneously increased [30]. The change in by-products can be seen in Figure 11 (alanine is the bacterial secretion).

### Effects of feeding 0.5 g/L trypsin on total proteins and amino nitrogen content

**Figure 12.**
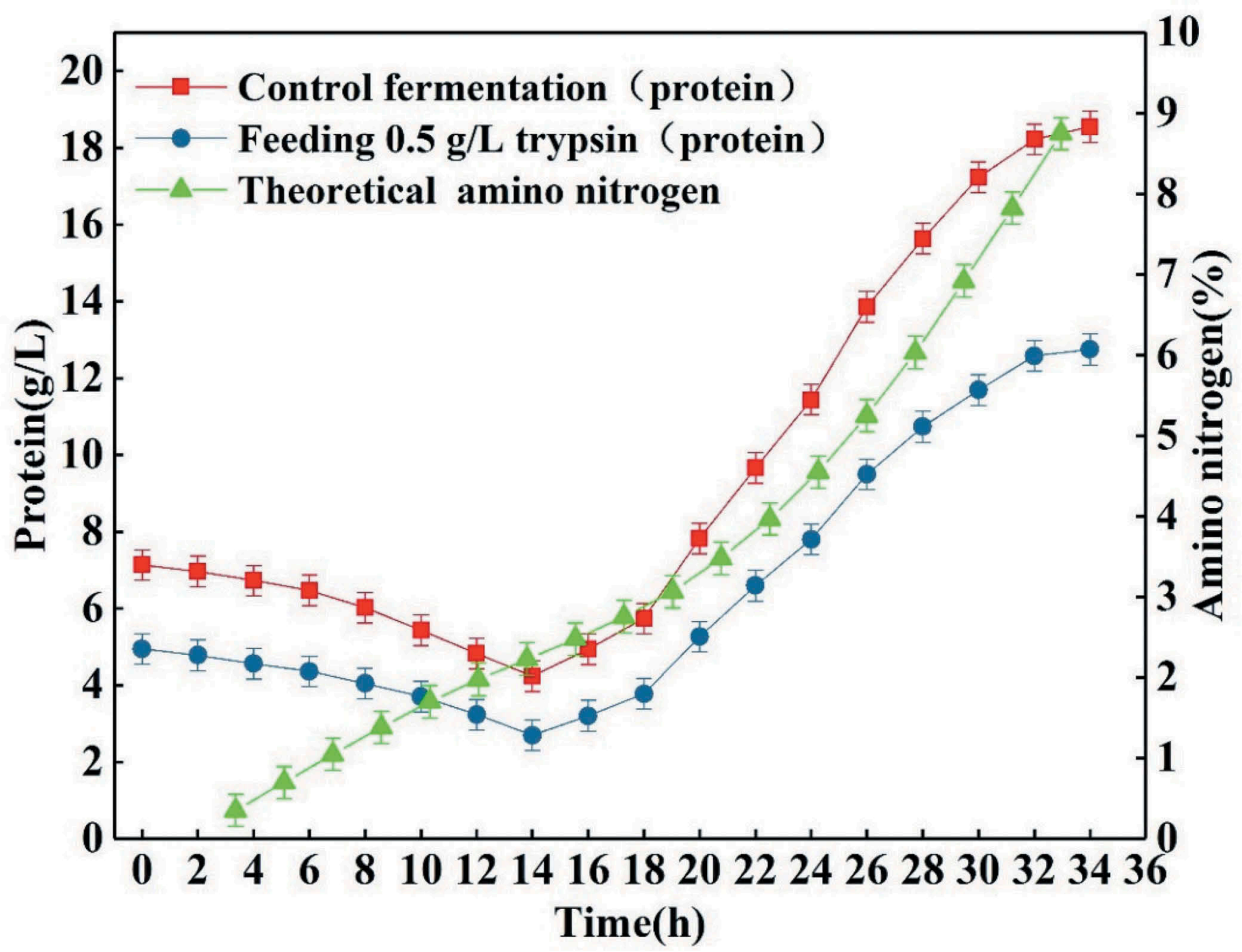
Effects of feeding 0.5 g/L trypsin on total protein and amino nitrogen content.

When feeding 0.5 g/L trypsin, the total proteins and amino nitrogen content were as shown in Figure 12. It can be seen that, with the enzymatic hydrolysis of proteins, the theoretical amino nitrogen (excluding nitrogen content in L-glutamic acid) content increases. However, further detection showed that the actual content of amino acids (excluding L-glutamic acid and by-product alanine) in fermentation broth was zero. The above data further proves that all the various amino acids obtained after hydrolysis of proteins have been utilized by the bacteria.

## Discussion

In this study, by adding different kinds of proteases during the fermentation to produce L-glutamic acid, the proteins in the culture medium and the proteins secreted by the cells were hydrolyzed to improve the efficiency of dissolved oxygen and improve the fermentation state. The experimental results showed that feeding 0.5 g/L trypsin at the initial fermentation stage was the best choice. Lin Z *et al*. [31] studied the digestion of proteins in the fermentation broth by adding immobilized trypsin in one administration and found that the total protein hydrolysis degree was only about 15.0%. In this study, although the fermentation conditions were not the optimal viability conditions for the protease, by adding trypsin, the hydrolysis of the total proteins in the fermentation broth was as high as about the 30.0%. These proteins were hydrolyzed into various amino acids, peptides and small molecular weight proteins that are used by the cells to promote the growth and metabolism of the cells. Çalık G *et al*. [32] studied the oxygen transfer efficiency in L-glutamic acid fermentation and found that the volume of dissolved oxygen coefficient K_L_a is difficult to enhance by just increasing the ventilation. In this study, the viscosity of the fermentation broth was reduced by hydrolysis of the proteins, so the dissolved oxygen efficiency was also improved, and the volumetric dissolved oxygen coefficient K_L_a could be greatly improved. In addition, the amount of ventilation and the amount of defoamer were also reduced. The overall reflection of the fermentation results is the increase of bacterial biomass, the rate of glucose consumption and the increase of L-glutamic acid production rate. The final L-glutamic acid production reached 177.0 g/L, which was 14.9% higher than the control fermentation (154.0 g/L), and the glucose conversion rate was 68.3%, which was 4.0% higher than the control fermentation (65.6%). Therefore, it is feasible to add trypsin to the growing medium to hydrolyze part of the proteins in the L-glutamic acid fermentation process and this has a great promotion effect on L-glutamic acid fermentation. This study only considered a portion of the single protease in L-glutamic acid fermentation process. Different ratios and kinds of various proteases and whether these also have good hydrolysis potentials and whether the proteases used in the fermentation process of other amino acids are similarly effective, need to be furtherly explored.

## Highlights

Decomposition of proteins during fermentation without causing bacterial damage; The hydrolyzed proteins can be used by bacteria to increase the utilization of nutrients; The decrease of proteins content is also beneficial to the subsequent separation and extraction of L-glutamic acid.
